# The Activation Pattern of Drug-Reacting T Cells Has an Impact on the Clinical Picture of Hypersensitivity Reactions

**DOI:** 10.3389/falgy.2022.804605

**Published:** 2022-02-21

**Authors:** Natascha Wuillemin, Barbara Ballmer-Weber, Christoph Schlapbach, Lukas Jörg, Daniel Yerly

**Affiliations:** ^1^Department of Rheumatology, Immunology and Allergology, University Hospital of Bern, Bern, Switzerland; ^2^Clinic for Dermatology and Allergology, Kantonsspital St. Gallen, St. Gallen, Switzerland; ^3^Department of Dermatology, University Hospital of Bern, Bern, Switzerland; ^4^Policlinic for Allergology and Clinical Immunology, University Clinic for Pneumology, University Hospital of Bern, Bern, Switzerland

**Keywords:** drug allergy, T cells, hapten concept, p–i-concept, drug reaction with eosinophilia and systemic symptoms (DRESS), maculopapular drug eruption, severe cutaneous adverse drug reaction

## Abstract

**Rationale:**

β-lactam antibiotics cause drug hypersensitivity reactions (DHR) with various clinical pictures from minor affections like maculopapular exanthema (MPE) and urticaria to severe cutaneous adverse reactions and anaphylaxis. Currently, two different reactivity patterns have been shown to initiate an immune reaction by activating T cells—the hapten concept and the pharmacological interaction with immune receptor (p–i) concept.

**Objectives:**

In this study, the relationship between the reactivity pattern of drug-reacting T cells of drug allergic patients and their clinical picture has been investigated.

**Findings:**

Drug-reacting T-cell clones (TCCs) were isolated from patients hypersensitive to β-lactams. Analysis of their reactivity pattern revealed an exclusive use of the hapten mechanism for patients with immediate reactions and for patients of MPE. In patients suffering from drug reactions with eosinophils and systemic symptoms, a severe DHR, analysis of isolated drug-reacting TCC identified the p–i concept as the unique mechanism for T-cell activation.

**Conclusions:**

The results show a shift from hapten pattern in mild allergic reactions to p–i pattern in severe life-threatening allergic reactions. They strongly argue against the current preclinical risk evaluation of new drugs based on the ability to form haptens.

## Introduction

Adverse drug reactions are a major cause of morbidity and mortality worldwide. Up to 30% of these adverse reactions are attributable to drug hypersensitivity reactions (DHRs). Most of these DHRs are mediated by the adaptive immune system and the severity of the symptoms extends from mild to life-threatening reactions. Not only anti-drug antibodies can be found in drug allergic patients, but also drug-reacting T cells (1). For delayed type DHR, drug-reacting T cells are thought to be the key player. Such T cells were observed in the skin lesions of drug-induced maculopapular exanthema (MPE) (2), in the blisters of severe Stevens Johnson syndrome (SJS) (3), and in internal organ lesions of drug reaction with eosinophils and systemic symptoms (DRESS) (4). In the case of immediate type hypersensitivity reaction, anti-drug IgE are essential for mast cells activation. Of note, isotype switch to IgE often requires the help of drug-reacting T cells, emphasizing their involvement in all types of DHR.

Antigen-specific stimulation of conventional T cells relies on T-cell receptor activation by peptides presented on HLA molecules. Currently, two fundamentally different concepts provide rational explanations of how a drug can initiate a DHR by activating human T cells—the hapten theory and the pharmacological interaction with immune receptor (p–i) concept. The hapten theory states that small chemical compounds bind covalently to endogenous proteins to form hapten-carrier complexes that are antigenic and induce T-cell responses. The p–i concept implies direct and reversible interactions of the drug with T-cell receptors and/or HLA molecules, mediated by Van der Waals interactions and hydrogen bonds. Such drug interactions with the receptors do not require antigen processing, resulting in a nearly immediate stimulation of specific T cells (5, 6). A typical example of haptens is penicillin G and its derivatives, which have been shown to covalently bind to lysine residues of serum proteins (7, 8). We previously documented that flucloxacillin, a penicillin-derivative, can stimulate T cells *via* the hapten and the p–i concept (9). Interestingly, the p–i concept was only observed in carriers of the HLA-B^*^57:01 molecule, who have an increased risk for flucloxacillin-induced liver injury. In the study of Wuillemin et al., it was surprising that a typical hapten molecule was able to stimulate T cells without the need of covalent modification.

In clinics, β-lactam antibiotics are the most frequent cause for drug allergies and the clinical picture is far from being uniform. Indeed, it can take the form of a typical immediate type I, IgE-mediated hypersensitivity reaction like urticaria, angioedema, or even anaphylaxis. Delayed type hypersensitivity reactions (type IV) can occur with a symptomatology spanning from mild MPE to life-threatening reactions like SJS, toxic epidermal necrolysis, or DRESS. Whereas these affections differ in the severity, in their organ tropism and in the extent of the tissue damage, they share drug-reacting T cells as the key players of their pathogenesis. Until now, the clinical relevance of the molecular activation mechanism of drug-reacting T cells has never been addressed and it is not known whether there is a link between the involved molecular activation mechanism and the clinical picture.

This study aimed at defining the role of the T-cell recognition pattern on the clinical picture. To this aim, we investigated the relationship between the reactivity pattern of β-lactam-reacting T cells found in the peripheral blood of allergic patients and the clinical phenotype. We expanded drug-reacting T cells from β-lactam allergic individuals, such as patients with typically IgE-mediated hypersensitivity reactions such as urticaria or anaphylaxis as well as patients with T-cell mediated reactions such as MPE and severe cutaneous adverse reactions (SCAR). The analysis of the drug recognition pattern of T cells revealed that the p–i activation mechanism was detected in severe reactions, whereas reactivity with hapten mechanism was observed in T cells from patients with type I or mild forms of type IV hypersensitivity reactions.

## Methods

### Allergic Patients

Eighteen β-lactam-allergic patients were enrolled in the study, such as patients with IgE-mediated hypersensitivity reactions including urticaria or anaphylaxis, patients with T-cell-mediated reactions such as MPE and SCAR. Patients with immediate type allergy were included if they presented a positive prick test, and patients with delayed-type allergy were included if they presented a patch test and/or a positive lymphocyte transformation test (LTT) to the β-lactam. All patients gave written informed consent prior to being enrolled in the study and the study was approved by the local ethical committee (KEK-189/13). All experiments have been carried out in accordance with the Declaration of Helsinki.

### The 5,6-Carboxyfluorescein Succinimidyl Ester-Based Proliferation Assay and T-Cell Clone Generation

After isolation by density gradient centrifugation, peripheral blood mononuclear cells (PBMCs, 4 × 10^6^ cells) were stained with 5,6-carboxyfluorescein succinimidyl ester (CFSE, 1 μM, Sigma Chemical, Buchs, Switzerland) and cultured for 7 days with the culprit β-lactam: either 500 μg/ml amoxicillin (AMX), 100 μg/ml cefuroxim, or 100 μg/ml ceftriaxone (Sigma Chemical) in 2 ml culture medium (CM) at 37°C and 5% CO_2_. Proliferation of drug stimulated CFSE-labeled cells was analyzed on a FACSCanto-I cytometer using FACS-Diva software (BD Biosciences, Basel, Switzerland) after staining with anti-CD3-PerCp-Cy5.5, anti-CD4-Pe-Cy7, and anti-CD8-APC-Cy7 antibodies (Biolegend, San Diego, CA, USA). Drug-reacting CD4^+^ and CD8^+^ T cells were isolated by flow cytometry-based cell sorting (FACSAria II, BD Biosciences) for CFSE^low^CD3^+^CD4^+^/CD8^+^ cells, respectively. Purity of cell populations was >98% after sorting. T-cell clones (TCCs) were generated by limiting dilution. Specificity of expanded TCC was analyzed by ^3^H-thymidine incorporation as described previously (10). TCCs without drug reactivity were discarded.

### T-Cell Stimulation With Drug and Distinction Between Hapten and p–i Reactivity

T cells were either stimulated with drug-pulsed antigen-presenting cell (APC) (APC^drug^) or with freshly added AMX (500 μg/ml), cefuroxime or ceftriaxone (100 μg/ml) in the presence of autologous APC (APC + drug). The latter is referred as “drug in solution.” EBV-B lymphoid cell line (EBV-BLCL) or PBMCs were used as autologous APC. For APC-pulsing, APC was incubated 14 h with drugs, either AMX (500 μg/ml), cefuroxime or ceftriaxone (100 μg/ml) at 37°C, followed by three washing steps to completely remove the unbound drug. The washing steps eliminate the drug in solution, i.e., meaning that the remaining drug is strongly (mostly covalently) bound to the macromolecules forming hapten in the APC. Altogether, APC^drug^ leads to stimulation of T cells *via* the hapten mechanism, whereas APC + drug lead to stimulation of T cells *via* p–i and the hapten mechanism. When the drug is added and maintained with the APC (APC + drug), the formation of a hapten cannot be excluded. Therefore, TCC reacting to haptens shows reactivity to APC^drug^ and APC + drug, whereas the p–i reacting TCC shows reactivity to APC + drug only and not to APC^drug^, because the drug needs to be present in the solution.

### Reactivity Testing Upon Drug Stimulation

After cloning and expansion, the reactivity of TCC toward the culprit drug was examined by flow cytometry by monitoring the upregulation of CD107a (PE, BioLegend) after a 6-h stimulation assay in the presence of monensin (6 μg/ml, Sigma) with the drug, as increase of CD107a surface expression rapidly correlates with TCC activation (11).

### Calcium Influx Assay

Calcium influx measurements were performed as described previously (5). Measurement was performed on a Synergy-4 instrument (BioTek, Highland Park, VT, USA). Baseline signal (F_0_) was recorded for 5 min before the addition of stimuli. Subsequently, fluorescence was measured for 70 min. The results are shown as normalized fluorescence (F/F_0_).

### Antigen Processing Inhibition

In order to block the antigen processing machinery and thus preventing the presentation of haptenized peptides on major histocompatibility complex (MHC) molecules, autologous PBMC, or EBV-BLCL were pre-incubated in CM containing the indicated concentrations of bortezomib (Velcade®, Janssen-Cilag AG, Baar, Switzerland) or chloroquine (Sigma), respectively, for 12 h at 37°C. Afterwards AMX (500 μg/ml), cefuroxime or ceftriaxone (100 μg/ml) was added for 14 h in order to pulse APC. The processing inhibition was performed with APC^drug^ or APC + drug as a stimulatory agent, as described in the T-cell stimulation section. If processing inhibition was performed with drug in solution, TCCs were previously incubated for 12 h with inhibitors in order to rule out self-presentation of drug. Activation of TCC was monitored by flow cytometry after a 6-h stimulation assay and staining for CD107a.

### Statistical Analysis

Statistical analyses were performed using GraphPad Prism 5 (GraphPad Software, San Diego, CA, USA). Comparisons were drawn using Wilcoxon matched pairs *t*-test or Mann–Whitney test. Each experiment was at least repeated twice. The *p*-values below 0.05 were regarded as statistically significant with ^*^p < 0.05, ^**^p < 0.01, ^***^p < 0.001 (CI 95%).

## Results and Discussion

### Drug-Reacting T Cells Can Be Expanded and Cloned From Peripheral Blood of DHR Patients

**Figure 1:**
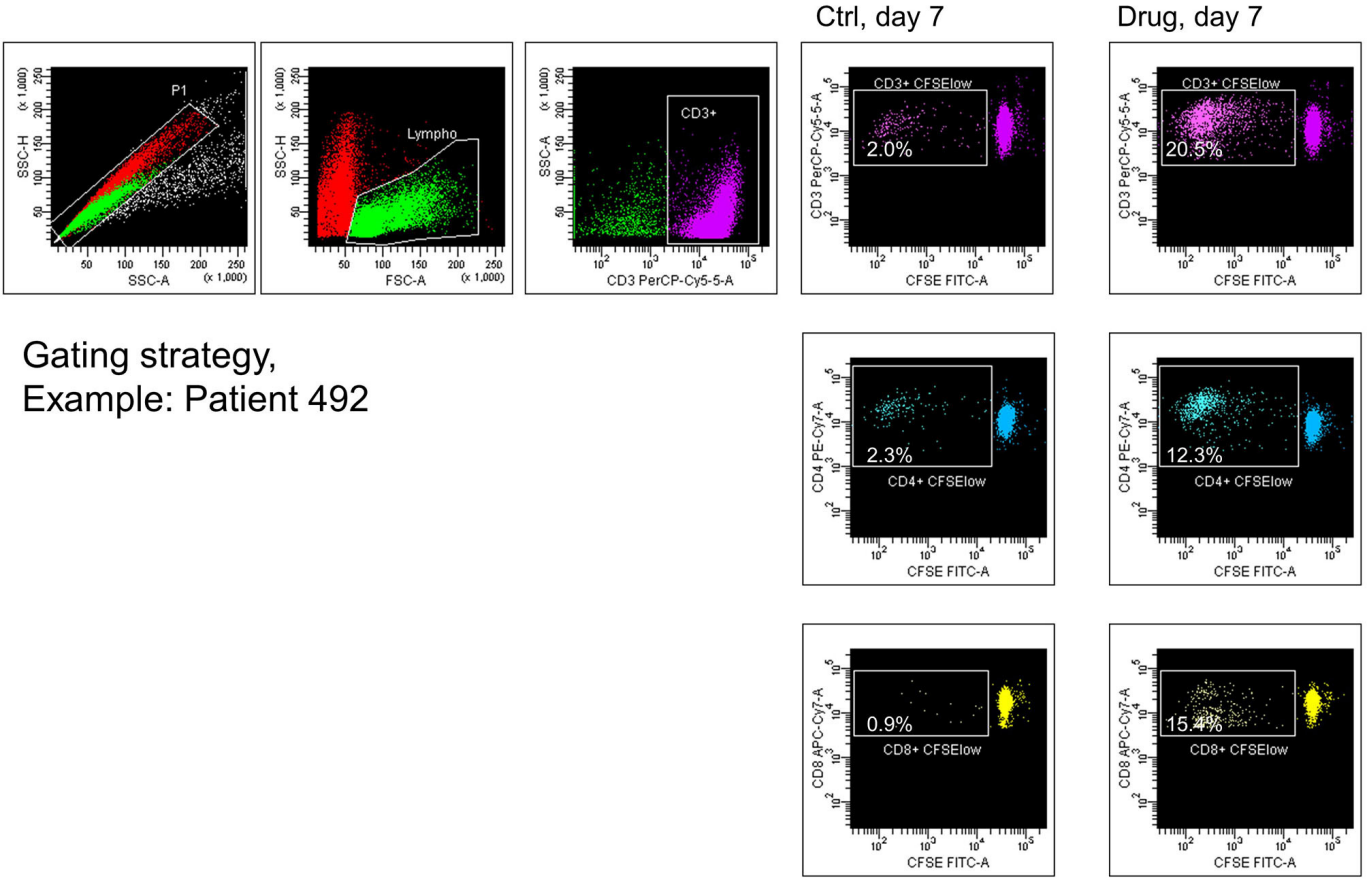
Proliferation, gating, sorting, and cloning of drug-reacting T cells. Peripheral blood mononuclear cells (PBMCs) from the patients of β-lactam hypersensitive (here, the example of patient 492) were labeled with 5,6-carboxyfluorescein succinimidyl ester (CFSE) and cultured in the absence (ctrl) or in the presence of the culprit β-lactam (drug). After 7 days, cell cultures were stained with anti-CD3, anti-CD4, and anti-CD8. Events were gated on singlet cells and lymphocytes, such as larger and more granular effector lymphocytes. CD3^+^ T cells showing low intensity of CFSE were sorted and directly cloned by limiting dilution. Percentages of CFSE^low^ in the CD3, CD4, and CD8 T cell compartment were determined.

In order to evaluate the impact of T-cell activation mechanisms on the clinical picture, patients with clearly defined types of DHR were recruited. They were categorized into 4 groups: (A) patients with immediate-type hypersensitivity reaction with symptoms as urticaria or anaphylaxis, (B) patients with delayed-type reaction with MPE, clinically diagnosed as an erythematous skin eruption with papules without signs of blistering, pustules, mucosal, or systemic involvement, (C) patients with DRESS, and (D) patients with SJS (Table 1). PBMCs were examined for T cells reacting to the culprit β-lactam antibiotic. In order to prevent *in vitro* priming, expansion of drug-reacting memory T cells was performed in the absence of recombinant interleukin 2 (IL-2). Briefly, PBMCs from drug allergic patients were cultured in the presence of the culprit β-lactam and after 7 days, proliferating T cells (CD3^+^), monitored by low content of CFSE, were sorted (Figure 1) and directly cloned by limiting dilution. To evaluate whether a drug-induced expansion of T cells had occurred *in vitro*; a stimulation index (SI) was calculated (Table 1). This SI is a ratio defined by the proportion of the proliferating cells in the conditions with the drug divided by the proportion of the proliferating cells in the control (medium only, background proliferation). A value of SI above 2 means that a drug-induced proliferation has occurred. In patients with type I hypersensitivity (group A), the number of patients with a drug-induced proliferation was quite low (1/4) and drug-reacting T cells were found mostly in the CD4^+^ compartment only. In the group of MPE (group B), all the patients reacted to the culprit drugs (SI > 2). The reacting cells were found in the CD4^+^ and CD8^+^ compartments. In the group C, with DRESS patients, drug reacting cells could be detected in the majority of the included individuals (3/4), also in both compartment CD4^+^ and CD8^+^ T cells (Table 1). Unfortunately, no drug-reacting T cells could be detected in the PBMC from the patients of SJS (group D). Even the cloning of the few dividing cells did not allow the identification of β-lactam-reacting T cells. Therefore, the group of patients of SJS could not be further investigated. Among the included individuals of the other groups, important differences of reactions intensities could be observed, with SI expending from 1 up to nearly 50. We assume that those differences could be due to several factors, like the delay between the reaction and the analysis as well as the intensity of the disease.

**Table 1:** Summary of proliferation data to beta lactam antibiotics.

	**Patients ID**	**Symptoms**	**Patient group**	**Culprit drug**	**SI**	**SI**	**SI**
					**CD3, cfse low**	**CD4, cfse low**	**CD8, cfse low**
1	515	Anaphylaxis	A	Amoxicillin	1.0	1.0	0.9
2	506	Anaphylaxis	A	Amoxicillin	3.7	3.5	1.0
3	700	Anaphylaxis	A	Cefuroxim	1.3	1.4	0.9
4	699	Anaphylaxis	A	Cefuroxim	1.2	1.3	2.0
5	31	MPE	B	Amoxicillin	11.3	9.3	20.7
6	492	MPE	B	Amoxicillin	10.8	5.2	25.7
7	491	MPE	B	Amoxicillin	25.8	49.4	14.8
8	466	MPE	B	Amoxicillin	7.0	8.0	0.0
9	244	MPE	B	Amoxicillin	10.0	11.0	0.0
10	689	MPE	B	Amoxicillin	5.2	5.4	4.8
11	687	MPE	B	Amoxicillin	2.1	2.3	0.5
12	698	DRESS	C	Amoxicillin	1.0	0.9	1.2
13	694	DRESS	C	Amoxicillin	8.7	7.9	0.2
14	697	DRESS	C	Ceftriaxone	3.2	3.5	1.4
15	696	DRESS	C	Ceftriaxone	18.4	19.8	17.1
16	695	SJS	D	Amoxicillin	0.9	0.9	0.5
17	404	SJS	D	Amoxicillin	1.0	1.1	0.9
18	460	SJS	D	Amoxicillin	0.9	1.0	0.8

*SI, stimulation index: ratio of CFSE^low^ population in drug stimulated condition normalized by the control (no drug)*.

In all groups, after expansion, drug-reacting colonies were identified by proliferation capacity or by upregulation of the CD107a marker, as described in the Methods Section. In total, we were able to expand 65 β-lactam-reacting TCC.

### T Cells From Patients With Immediate Hypersensitivity Reactions Recognize Amoxicillin According to the Hapten Concept

Distinction between drug recognition by the hapten or by the p–i concept was essentially made by analyzing the stability or the lability of drug binding on APC. In contrast to non-covalently bound drugs, haptens cannot be removed from APC by extensive washing steps. Furthermore, the presentation of haptenized peptides on HLA molecules requires processing. To investigate the reactivity pattern of the TCC, several activation conditions, and their impact on T-cell activation were analyzed by flow cytometry. β-lactam-reacting T cells were either stimulated with drug in solution (APC + drug) or drug-pulsed APC (APC^drug^). In summary, hapten reactivity was defined as stimulation with pulsed APC and p–i reactivity as stimulation by drug in solution but not with pulsed APC. Of note, in the CD107a upregulation assay, T cells reacting to hapten can react to the condition “drug in solution” as well, since the stimulation assay went on for 6 h, sufficient incubation time allowing the formation of haptenized antigens and their presentation. Therefore, reactivity of hapten reacting TCC can be found in APC^drug^ as well as in APC + drug, whereas p–i-reacting TCCs are found only when the drug in solution is present (APC + drug).

**Figure 2:**
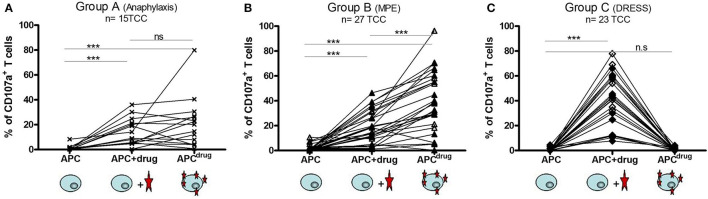
T cells from type I hypersensitivity and maculopapular exanthema (MPE) react according to hapten mechanism and T cells from drug reaction with eosinophils and systemic symptoms (DRESS) react according to p–i concept. The stability/lability of the antigen stimulating complex was analyzed for TCC from patients from group A, B, and C. Each T-cell clone (TCC) was stimulated separately with autologous EBV-BLCL [antigen-presenting cell (APC)] (for background activity), with EBV-BLCL previously pulsed overnight with the culprit β-lactam (APC^drug^), and with EBV-BLCL and β-lactam solubilized in CM (APC + drug). After 6 h, upregulation of the activation marker CD107a was analyzed by flow cytometry. An increase in the intensity of CD107a expression demonstrates the activation of the TCC. TCC reacting according to p–i concept are meant to react to APC + drug only, whereas TCC reacting according to the hapten mechanism are meant to react to APC^drug^ and/or APC + drug. **(A)** 15 TCC from 3 patients were analyzed, 12 showing reactivity for amoxicillin and 3 for cefuroxime. **(B)** 27 TCC from 4 patients (MPE) were analyzed, all showing reactivity to amoxicillin. **(C)** 23 TCC from 3 patients of DRESS were analyzed, with 6 reacting to amoxicillin and 17 to ceftriaxone. The plots with single reactivity (amoxicillin, cefuroxime, or ceftriaxone) are shown in the Supplementary Figure.

In patients from the group A (*N* = 4), TCC were found to react to haptenized antigens only. All generated TCC reacted to pulsed APC (Figure 2A). These results were expected, as the β-lactam ring can open under physiological conditions and can form adducts with proteins. Type I hypersensitivity are mediated with by antibodies (IgE), which were shown to follow the hapten mechanism (8).

### The Majority of TCC From the Patients of MPE Reacted to Haptenized Antigens

In patients of the group B (*N* = 7), the AMX-reacting TCC was showed to recognize the haptenized antigens as well. Indeed out of 27 generated TCC, two could not be activated by APC^drug^ (hapten), but only by APC with drug in solution (Figure 2B). These data confirm the relevance of hapten formation in MPE. Recently, a study by Amali et al. revealed the presence of anti-drug IgG in MPE to piperacillin, which was shown to recognize haptenized protein adducts (piperacillin-BSA). Anti-drug antibodies are in line with the hapten theory, as β-lactam is thought to be too small to be sufficiently antigenic (12). Together with hapten-reacting T cells, it appears likely that macromolecules haptenized by β-lactam constitute the main immunogenic determinant in MPE.

### Drug-Reacting T Cells From the Patients of DRESS React Exclusively According to the p–i Concept

For the third group of patients (C, *N* = 4) suffering from DRESS, all TCC reacting to AMX or ceftriaxone from the three patients exhibited the same pattern: they did not react to β-lactam-pulsed APC, but to drug in solution, indicating an exclusive p–i reactivity pattern (Figure 2C). These results show the association between the p–i mechanism and severe delayed-hypersensitivity reactions. In comparison with the two other clinical pictures (anaphylaxis and MPE), where antibodies also play an important role in the pathomechanism (12), the occurrence of anti-drug antibodies in DRESS and other SCARs needs to be explored. In anaphylaxis and MPE, drug-reacting T cells following the hapten mechanism were associated with IgE and IgG antibodies, respectively. In the case of DRESS, where haptenized macromolecules do not represent the antigenic determinant, the presence and the conceivable function of anti-drug antibody are questionable. Antibody-dependent activation is usually based on cross-linking of antibodies. This requires the presence of a larger molecule that is able to bind to two antibodies simultaneously. It is, however, difficult to imagine, how antibodies could be cross-linked by labile small molecules that are not covalently bound to proteins.

### Immediate Activation of TCC From the Patients of DRESS by Drug in Solution

**Figure 3:**
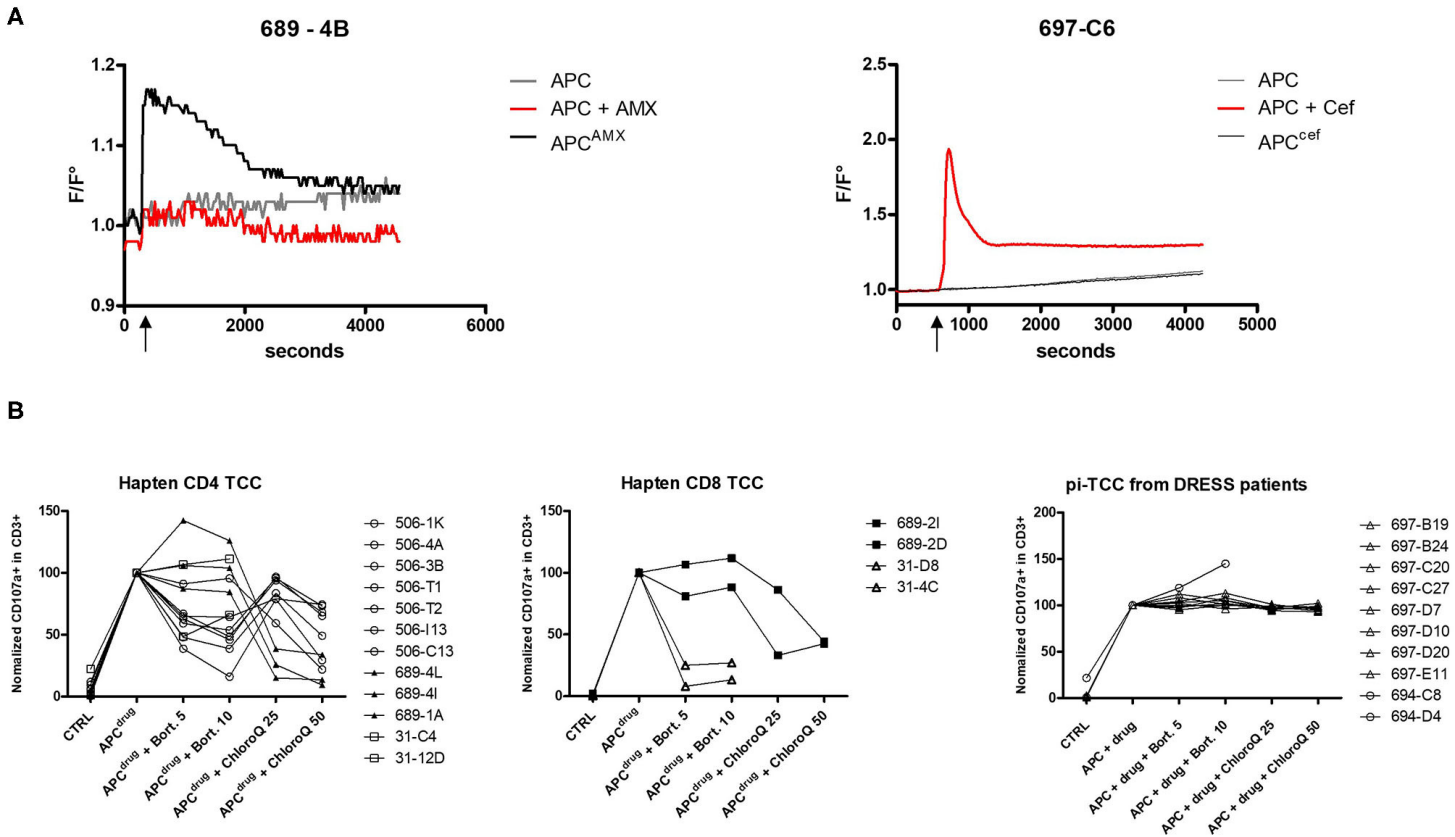
Comparison of hapten and p–i features of TCC reacting to β-lactams. **(A)** Intracellular calcium concentration (represented by F/F_0_ ratio) in TCC was measured by fluorescence. After 5 min (600 s) of baseline measurement, β-lactam-pulsed EBV-BLCL and EBV-BLCL together with drug in solution was separately added. The increase of calcium concentration was monitored for 60 min. The left plot shows an example of a TCC reacting according to the hapten mechanism (patient 689 suffering from MPE) and the right plot shows an example of a TCC reacting to ceftriaxone according to p–i from a patient of DRESS (697). **(B)** The involvement of the antigen processing machinery has been investigated by adding bortezomib and chloroquine during APC pulsing and TCC stimulation. TCC activation was measured by CD107a upregulation with flow cytometry and TCC activation with pulsed APC (APC^drug^) was normalized to 100%. Measurements were repeated at least twice.

So far, T-cell response to β-lactam was analyzed 6 h after addition of the culprit drug. To characterize the kinetics of T-cell activation early after drug stimulation, we performed calcium influx measurements. After 5 min of baseline measurements, drug-pulsed APC was added to the TCC (Figure 3A). None of the tested TCC derived from the patients of DRESS reacted to drug-pulsed APC. The activation of TCC derived from the patients of DRESS required the addition of drug in solution, which resulted in an immediate activation reaching its maximum activation level after 2 min and 10 s. This immediate activation kinetic was consistently observed among all analyzed TCC derived from the patients of DRESS.

### Delayed Activation of TCC From the Patients of MPE by Drug in Solution

In contrast to TCC derived from the patients of SCAR, TCC from groups A and B showed an increase in intracellular calcium upon stimulation with pulsed APC (Figure 3A). The addition of AMX-pulsed APC resulted in an immediate calcium influx, reaching its maximum within 2 min, whereas they did not react upon the stimulation with drug in solution during the whole duration of the assay. These results imply the requirement of a time-dependent activation step to render immunogenic AMX.

### Stable Presentation of β-Lactams Does Not Require the Antigen Processing Machinery

The stable presentation of β-lactams and the time dependence in the patients of MPE and anaphylaxis suggest a hapten mechanism of β-lactam-presentation. Due to their antigenic characteristics, haptens have to be processed by the processing and presentation machinery to be suitably presented. On the one hand, we investigated the role of the endogenous processing pathway by inhibiting the proteasome with bortezomib. On the other hand, the involvement of the exogenous pathway was assessed by inhibiting the acidification of the endosome with chloroquine. CD4^+^ and CD8^+^ TCC with hapten reactivity from three patients of groups A and B were tested (Figure 3B). In all analyzed TCC, one of the two pathways was critical for antigen presentation. Surprisingly, CD4^+^ T cells did not rely uniquely on the exogenous pathway. Seven out of 12 TCC exhibited reduced reactivity when the proteasome was inhibited. The involvement of the endogenous pathway for CD4^+^ TCC suggests a non-canonical MHC class I restriction, as has been observed in the drug hypersensitivity field (13). Due to insufficient growth of primary TCC, a complete HLA restriction analysis could not be performed, but the analysis of two TCCs confirmed this assumption, implying a presentation by MHC class I for CD4^+^ TCC (data not shown). Inversely, two CD8^+^ TCCs from a patient with MPE revealed an involvement of the exogenous antigen processing pathway. TCCs from two patients of DRESS were also assessed for antigen processing. Neither proteasome inhibition nor the addition of chloroquine was able to reduce the reactivity of these TCCs. Together with the immediate activation pattern and with the activation by a drug in solution uniquely, the independence of the processing confirms the p–i pattern of T cells found in the patients of DRESS.

In summary, we could demonstrate a correlation between the clinical phenotype of allergic reaction and the mechanism of drug presentation by reacting T cells. The hapten mechanism is responsible for type I DHR and MPE. However, in patients with DRESS β-lactams are activating T cells by the p–i mechanism. Of course, a higher number of reacting patients with severe reactions would consolidate our conclusions. The fact that severe cases of drug hypersensitivity like DRESS are mediated exclusively by the p–i-reacting TCC questions the quality of the currently used antigenicity prediction models which is based on the ability to build haptens only.

## Data Availability Statement

The raw data supporting the conclusions of this article will be made available by the authors, without undue reservation.

## Ethics Statement

The studies involving human participants were reviewed and approved by Ethikkommission, Kanton Bern, Schweiz. The patients/participants provided their written informed consent to participate in this study.

## Author Contributions

DY: conceptualization and supervision. DY and NW: methodology, investigation, and writing. NW, BB-W, CS, and LJ: resources. All authors contributed to the article and approved the submitted version.

## Funding

This work was financially supported by the Ulrich-Müller-Gierok Foundation in Bern (Switzerland).

## Conflict of Interest

DY was employed by the University Hospital of Bern, at the time of the study. Currently he is employed by ADR-AC GmbH, a private laboratory. The remaining authors declare that the research was conducted in the absence of any commercial or financial relationships that could be construed as a potential conflict of interest.

## Publisher's Note

All claims expressed in this article are solely those of the authors and do not necessarily represent those of their affiliated organizations, or those of the publisher, the editors and the reviewers. Any product that may be evaluated in this article, or claim that may be made by its manufacturer, is not guaranteed or endorsed by the publisher.
