# Supplementary figures and images for: The Activation Pattern of Drug-Reacting T Cells Has an Impact on the Clinical Picture of Hypersensitivity Reactions
Source: Front Allergy. 2022 Feb 21;3:804605. doi: 10.3389/falgy.2022.804605 (PMC8974706; doi:10.3389/falgy.2022.804605)

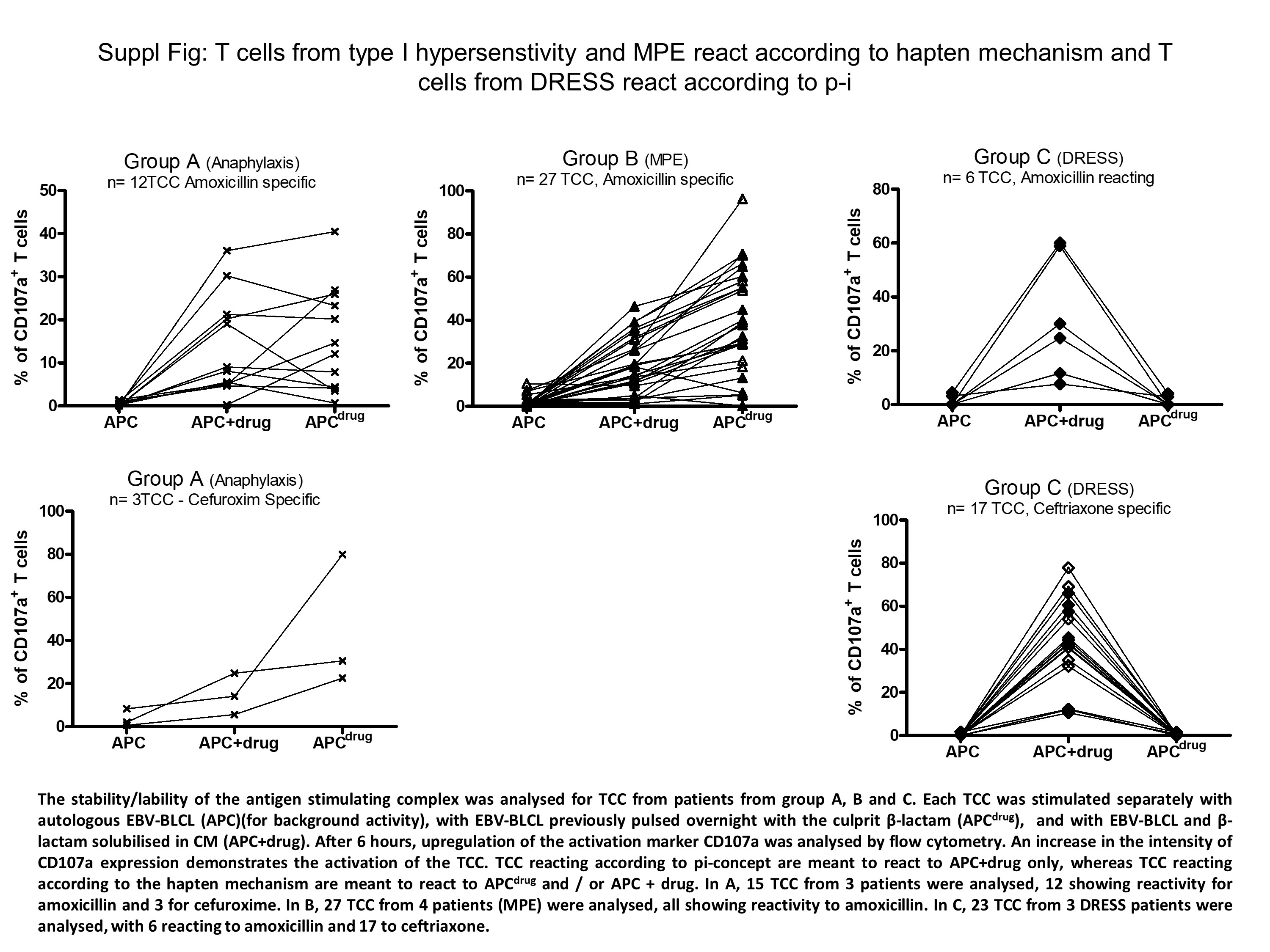

Supplement: Supplementary file 1 [file Image_1.JPEG]
